# Seawater Incursion Events in a Cretaceous Paleo-lake Revealed by Specific Marine Biological Markers

**DOI:** 10.1038/srep09508

**Published:** 2015-05-07

**Authors:** J. F. Hu, P. A. Peng, M. Y. Liu, D. P. Xi, J. Z. Song, X. Q. Wan, C. S. Wang

**Affiliations:** 1 State Key Laboratory of Organic Geochemistry, Guangzhou Institute of Geochemistry, Chinese Academy of Sciences, Guangzhou. 510640, P R China; 2 State Key Laboratory of Biogeology and Environmental Geology, China University of Geosciences, Beijing 100083, China

## Abstract

Many large paleo-lakes in North China were formed after the Triassic Era. Seawater incursion events (SWIEs) in these lakes have been extensively discussed in the literature, yet lack reliable methodology and solid evidence, which are essential for reconstructing and confirming SWIEs. The present study employs specific marine biological markers (24-*n*-propyl and 24-isopropyl cholestanes) to trace SWIEs in a dated core taken from the Songliao Basin (SLB). Two SWIEs were identified. The first SWIE from 91.37 to 89.00 Ma, was continuous and variable but not strong, while the second SWIE from 84.72 to 83.72 Ma was episodic and strong. SWIEs caused high total organic carbon (TOC) and negative δ^13^C_org_ values in the sediments, which were interpreted as an indication of high productivity in the lake, due to the enhancement of nutrient supplies as well as high levels of aqueous CO_2_, due to the mixing of alkaline seawater and acidic lake water. The SWIEs in SLB were controlled by regional tectonic activity and eustatic variation. Movement direction changes of the Izanagi/Kula Plate in 90 Ma and 84 Ma created faults and triggered SWIEs. A high sea level, from 90 to 84 Ma, also facilitated the occurrence of SWIEs in SLB.

No major marine deposits have occurred in Northern China since the Triassic Era and more than 20 large paleo-lakes such as Songliao, Bohai, Biyang, Subei and Qianjiang—have subsequently formed (during the Cretaceous and Tertiary Eras). The presence of widespread and massive petroleum deposits in these lacustrine sediments provide insights into the formation mechanisms of these lakes. From the 1950s onwards the relationship between these, and other, marine deposits, has attracted the attention of scientists, who believe that a detailed study on this topic will enhance understanding of tectonics and lacustrine sedimentation that have taken place since the Cretaceous Era.

Geologists, who noted that SWIEs frequently occur in the Songliao Paleo-lake, have proposed that SWIEs are closely related to the development of oil-source rocks in this lake. Since 1950, a considerable body of evidence—including the identification of marine algal fossils such as Dinophyceae^1^, planktonic foraminifera^2^, shark teeth^3^, and biological marker dinoflagellate steranes^4^ in the strata of the Songliao Basin —suggested deposition conditions with a strong marine influence. However, some controversy concerning this theory, still remains. The planktonic foraminifera and shark teeth have either been dramatically altered by early diagenesis and/or lacustrine water which may possibly be due to low pH values, relative to that of seawater. Diagenesis also modifies the morphology of Dinophyceae (during the process of oil generation, when the geo-temperature reaches 80°C). This is indeed the case for Dinophyceae in SLB as the referectance of vitrinite in the strata is more than 0.8%. Biological markers (dinoflagellate steranes) are not specific to the marine environment: they can also be found in brackish lacustrine sediments. For this reason, there is a need to obtain new specific evidence for SWIEs.

Biological markers, or “molecular fossils” extracted from ancient sediments and petroleum deposits have been widely used for the assessment of depositional environments, organic faces, or types of organic input^5^,^6^,^7^. Sterane fossilized sterols are important proxies for the reconstruction of paleo environments. Sterols containing 30 carbons are rare and have been reported to occur primarily in marine sponges^8^, pelagophyte algae^9^ and dinoflagellates^10^. Correspondingly, a series of C_30_-sterane epimers has been successfully established as a widespread fossil marker for marine organic input into sedimentary rock and petroleum, and to distinguish marine sediments and oils from non-marine counterparts^11^. Among them, C_30_ steranes (with an *n*-propyl substituent at C-24) are exclusively produced by marine pelagophyte algae^12^, while C_30_ steranes (with an isopropyl at C-24) are only produced by marine demosponges^13^. Dinosteranes (4,23,24-trimethylcholestanes) are uniquely derived from dinoflagellates^14^ and, in most cases, are indicators of a marine environment. The appearance of these sterane compounds, especially *n*-propyl and isopropyl C_30_ steranes in lacustrine sediments, can therefore be regarded as confirmation of marine organic input into lakes, i.e. as the tracer for SWIEs.

Sterane biological markers are derived from algal cell membranes. They are resistant to biodegradation in the water column and could survive in the sedimentary rocks experienced the diagenesis. Moreover, since steranes are readily detected in the sediments at very low concentrations (such as 10^−12^ g per gram of rock) they can potentially be used as a proxy for certain environmental conditions. All of these advantages suggest that marine-origin steranes can be used as a tracer, to indicate seawater incursions into lakes.

In this paper we present the quantitative distributions of these C_30_ sterane biomarkers in the first composited core, SK1, taken from the SLB, under the framework of the International Continental Scientific Drilling Program. Based on high-precision geochronology, these constitute a continuous nearly-30-Myr-long deposition, from the Turonian to Danian during the late Cretaceous Era, which spans the K/Pg boundary, extending into the Paleocene.

The chronostratigraphic framework of the SK1 core—described by Deng et al. (2013)^15^—is constrained by high-quality SIMS U-Pb zircon radiometric ages and by magnetostratigraphy. Four ^206^Pb/^238^U ages were determined and eleven local magnetozones have been identified as Chrons C34N to C28N^15^. Based on the natural gamma-ray log, thorium log, and magnetic susceptibility data from this core, an astronomical timescale was established by calibrating extracted 405-kyr cycles to the La2010a astronomical solution. The astronomical timescale analysis can provide high-resolution estimates of the age of each depth, at an error of <1000 yr^16^,^17^.

24-*n*-Propyl cholestanes and 24-isopropyl cholestanes can be isolated from core samples by means of extraction of dichloromethane and methanol, followed by purification by column chromatography. The concentrations of these compounds are measured by GC-MS/MS, with an internal standard of deuterated sterane.

The absolute abundances of extractable 24-*n*-propyl cholestanes and 24-isopropyl cholestanes are 0–15.7 and 0–2.93 μg·g^−1^ in the SK1 core, respectively (Fig. 1). The temporal distributions of these C_30_ sterane biomarkers indicate that marine organic matter input to the gigantic ancient freshwater lake (i.e. SWIEs) mainly occurred in the Upper Cretaceous Qingshankou and Nenjiang Formations (Fig. 1). SWIEs in the Qingshankou stage started from 91.37 Ma and terminated in 89.00 Ma, with a time span of 1.37 Ma, while, SWIEs in the Nenjiang stage were triggered in 84.72 and ended in 83.72 Ma, with a narrower time span, of 1.00 Ma.

The exact patterns of these biomarkers however suggest that the extent and process of SWIEs in the Nenjiang Formation was different to that in the Qingshankou Formation.

The concentrations of C_30_ steranes in samples from Qingshankou Formation are low for most of the time of the SWIEs, with the exception of samples with the age of 90.40 Ma. 24-Isopropylcholestane and 24-*n*-propylcholestane increased gradually from the top of Member 4 of the Quantou Formation (K_2*q*_^4^) to the Qingshankou Formation, reaching peak values at the top of Member 1 of the Qingshankou Formation (K_2*qn*_^1^), then decreasing gradually (Fig. 1b). The occurrence of these compounds indicates that modicus marine pelagophyte algae and demosponges entered the Songliao paleo-lake as marine-water injections. The concentration curve of C_30_ steranes (Fig. 1) suggests that at most time-stages the seawater incursion was continuous and variable, but not strong. The sulfur geochemical study of the sediments also supports the suggestion that the SWIE was not strong in the Qingshankou Formation^18^. A dramatic increase of C_30_ steranes in 90.40 Ma implies a massive incursion of seawater, possibly due to tectonic and geological events occurring in this region at this time.

By contrast, these C_30_ steranes were more abundant, and exhibited episodic patterns, in the Nenjiang Formation, indicating that the marine incursions were stronger and that there were seven main transgressive interludes in Member 1 and Member 2 of the Nenjiang Formation (K_2*n*_^1+2^, Fig. 1c). A detailed examination of the data points indicates that in some samples C_30_ sterane concentrations dropped to zero. These were interspersed between samples with high- C_30_ sterane concentrations. This indicates periodic closures of the seaway into the lake, during which times there was a cessation of seawater incursions. This observation is supported by δD of *n*-alkanes of those samples^19^. Extreme negative δD values of *n*-alkanes indicate that surface water, in which algae lived, was severely diluted by freshwater, indicating that the seaway had closed and that the SLB could store a higher proportion of freshwater.

The timing of the appearance of pelagophyte algae and demosponge biomarkers corresponds well with the positive excursion of the TOC (Fig. 2), implying that SWIEs may relate to the formation of organic-rich petroleum-source rocks in the SLB. In the Qingshankou Formation, the TOC increased abruptly at the top of K_2*q*_^4^ and remained at a relatively stable high TOC stage until Member 2 and Member 3 of the Qingshankou Formation (Fig. 2a, K_2*qn*_^2+3^), while TOC in the Nenjiang Formation (Fig. 2b) exhibited episodic patterns as was also the case for C_30_ steranes. One possible explanation for the high TOC in sediments is that a massive quantity of marine algae entered the paleo-lake when marine transgressions occurred. If we compare the δ^13^C_org_ of Qingshankou and Nenjiang oil-source rocks with those of sediments of Oceanic Anoxic Event 2 (OAE2) and Oceanic Anoxic Event 3 (OAE3), which are of similar ages, we find that the former is much depleted in ^13^C. This implies that high TOC in the source rocks may not only be due to marine algal input. Another possibility is that primary productivity increased in the lake during the SWIEs, which were mainly caused by tectonic activities. Such a scenario would enhance the weathering of mountains surrounding the lake and consequently increase nutrition supplies. The δ^13^C_org_ values in source rocks, mentioned in the following text, confirm that lacustrine organic matter is more significant.

δ^13^C_org_ values of source rocks in SWIEs indicate a negative excursion (Fig. 2), which differs from the positive excursions of OAE2 which occurred in marine conditions. A rational explanation for the negative excursion is that aqueous CO_2_ may increase by the mixing process when alkaline seawater enters a relatively acidic lake. A high CO_2_ concentration will result in the deletion of ^13^C photosynthetic products. The acidic nature of lake water in the Songliao paleo-lake is evidenced by the very thin shells of ostracods living in this lake during that time^20^.

The marine transgressions were mainly controlled by area tectonism. Songliao Lake was located in East Asia and the movement of the Izanagi/Kula Plate (IKP: Fig. 3)—one of Paleo Pacific Ocean Plates—strongly influences the morphology of the lake. According to reconstruction results, the Paleo Pacific Ocean Plate, IKP, suddenly changed direction, from N35°W to N15°W to N0°W, corresponding to the ages of 90 Ma, 84 Ma and 71 Ma, respectively^21^,^22^. The shifts of IKP movement create sinistral slip fault systems, allowing seawater to enter Songliao Lake. The turning times of IKP movement directions, in 90 Ma and 84 Ma, coincide well with those of SWIEs in SLB. Independent studies on the Tan-Lu fault system also show that it opened and deepened towards the northwestern Palaeo-Pacific^21^,^22^,^23^, which created the passage for marine water influx into the gigantic paleo-lake.

The SWIEs were also interrelated with eustatic changes. The global sea level was high in OAE2 and OAE3 periods and quickly descended after OAE3, due to the complete opening of the Atlantic Ocean^24^,^25^,^26^. Sea levels in OAE2 and OAE3 may have been 250–300 m higher than it is today^27^. A high sea level plus tectonic movement caused SWIEs in SLB. IKP activity in 71 Ma did not create SWIEs, possibly due to the low sea level, or uplifting of mountains surrounding the SLB during that time.

In conclusion, marine-specific biological markers are useful for the reconstruction of SWIE history in paleo lakes. As mentioned above, many large lakes have appeared in Northern China since the Triassic Era. Seawater incursions into those lakes have been extensively discussed in the literature but reliable evidence is needed to confirm these events, as well as appropriate methodologies for reconstructing detailed processes. The present paper provides an example of such a study, the purpose of which is to provide further insights into prospective biological markers aimed at solving these problems.

## Methods

### TOC and carbon-isotope analysis

Solvent-rinsed core rock fragments and cuttings were ground to 80 meshes after which 5% hydrochloride was added to a portion of each sample, to eliminate inorganic carbonates. To determine the percentage weight of organic carbon and δ^13^C_org_, bulk samples were analyzed: firstly by an elementary analyzer and then by an elementary analyzer combined with an isotopic-ratio mass spectrometer (see Supplementary 1).

### C_30_ steranes analysis

A portion of rock powder (20–30 g) was extracted, together with a mixture of dichloromethane and methanol (9:1, v/v), by Soxhlet reflux for 48 h. Asphaltenes were precipitated from the resulting organic extracts (bitumens) using *n*-hexane. The maltenes (*n*-hexane solubles) were then fractionated by silica gel/Al_2_O_3_ adsorption chromatography, eluting successively with hexane, hexane/CH_2_Cl_2_ (v/v: 1:1) and CH_2_Cl_2_/CH_3_OH (v/v: 2:1), to yield saturated hydrocarbons, aromatic hydrocarbons and polar fractions, respectively.

GC–MS analyses of saturated hydrocarbon fractions were performed on a Thermo ULTRA/DSQII equipped with TRACE GC and a DB-1MS-coated capillary column (60 m × 0.32 mm i.d., 0.25-μm film thickness) using He as carrier gas. Hopane and sterane biomarkers were analyzed by a Thermo TSQ Quantum XLS equipped with Trace GC ULTRA and a DB-1MS-coated capillary column (60 m × 0.32 mm i.d.; 0.25-μm film thickness) (scan time: 0.25 s; collision energy (CE): 10 v). The following levels of m/z were selected: parent and daughter ions: m/z 414 to 217 transition for 24-propylcholestanes: m/z 414 to 98 transition for dinosteranes. The GC oven was programmed at 100°C (1 min), heated to 220°C at 4°C/min, further heated to 300°C at 2°C/min, and held at final temperature for 20 min.

Fifty nanograms of deuterated C_27_ sterane standard [d4-ααα (20R)-Cholestane] was added as an internal standard to quantify the sterane biomarker content. Yields assume equal mass spectral response factors between analytes. Compound quantification was performed by peak area integration of m/z 217 in the extracted ion chromatogram for 24-propylcholestanes. Analytical errors were estimated to be lower than 5% for target compounds.

## Author Contributions

J.F.H. and P.A.P. equally contributed to the writing of the manuscript with additional support from D.P.X. M.Y.L. contributed significant data (TOC, isotopes, C_30_ steranes analyses). J.Z.S. collected the samples analyzed and did some preliminary work on it. C.S.W. collected the core and contributed to the ideas of the manuscript. X.Q.W. contributed to the ideas and writing of the manuscript, and financially supported part of the project.

## Supplementary Material

Supplementary Information Supplementary

## Figures and Tables

**Figure 1:**
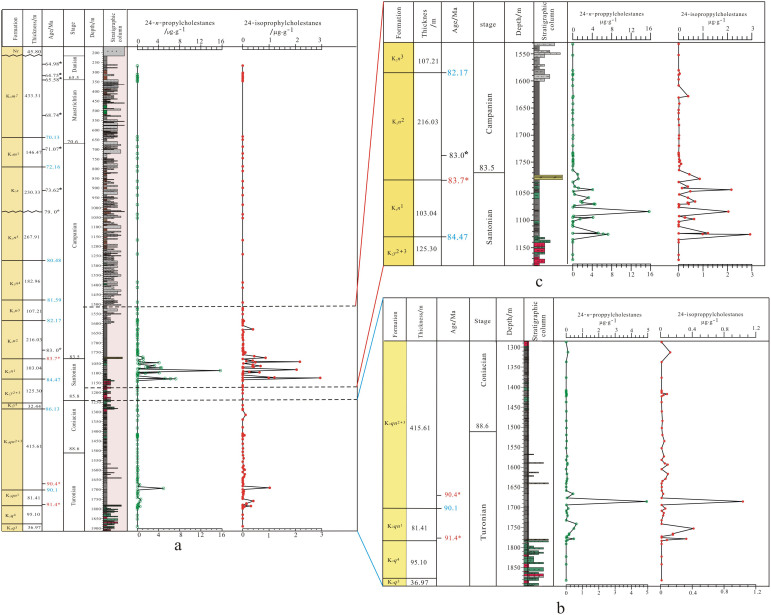
Stratigraphic column of Upper Cretaceous recovered from the SK1 core in the Songliao Basin, with representative formation, stage, lithology as well as geochronological constraints (Modified after Wang et al., 2013a (Ref. 28)). (a): Stratigraphic distributions of specific C_30_ steranes for whole SK1 core; (b): Enlarged view of stratigraphic distributions of specific C_30_ steranes for the Qingshankou Formation; (c): Enlarged view of stratigraphic distributions of specific C_30_ steranes for Member 1 and Member 2 of the Nenjiang Formation. Absolute dates in red * are SIMS zircon U-Pb ages, and dates in ☆ are paleomagnetic interpolated ages (Ref. 15). 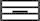 mudstone, 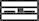 silty mudstone, 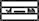 muddy siltstone, 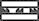 fine sandstone, 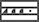 sandstone, 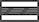 oil shale.

**Figure 2:**
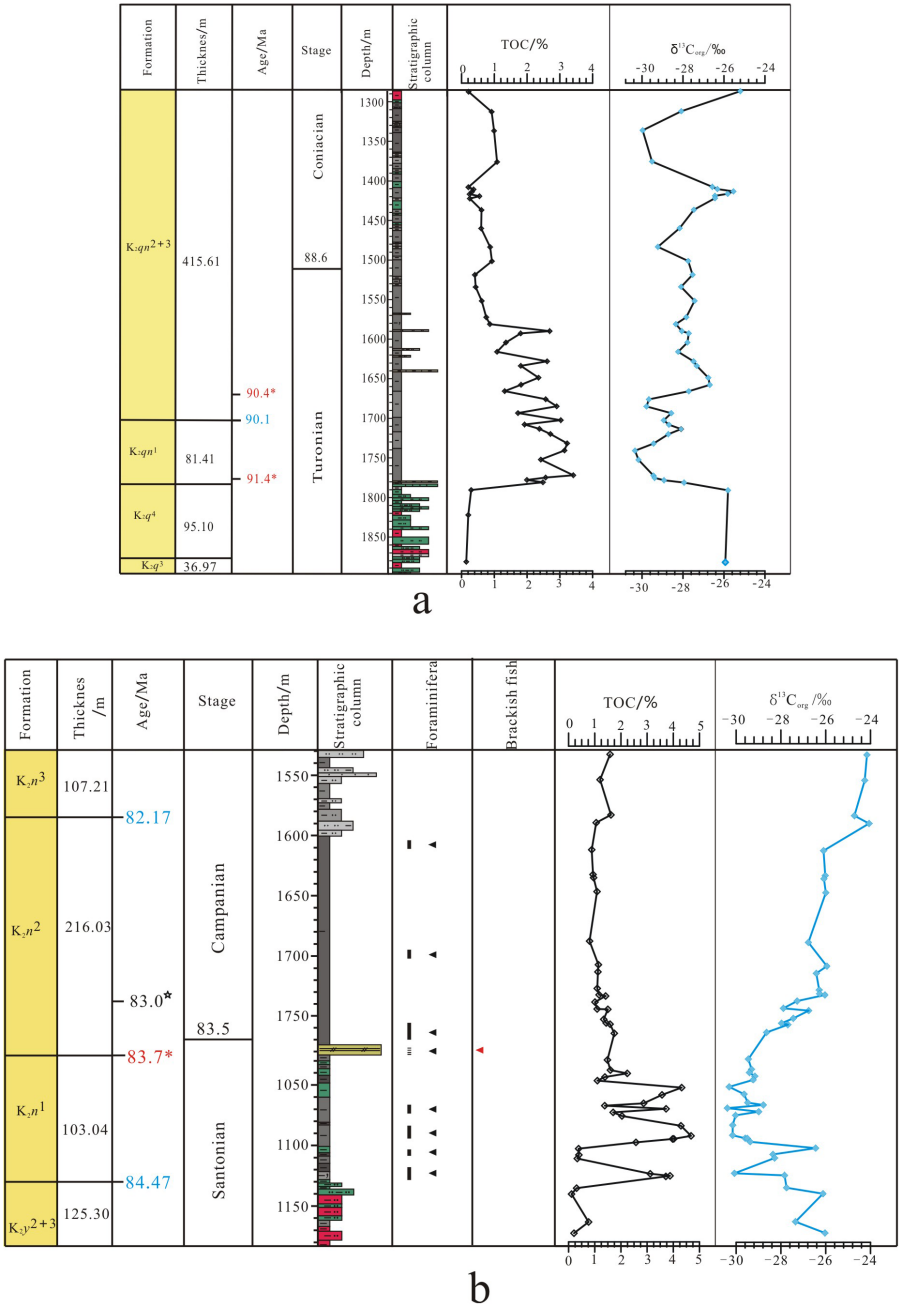
Stratigraphic distributions of TOC and δ^13^C_org_. (a): for Qingshankou Formation; (b): for Member 1 and Member 2 of the Nenjiang Formation. ◂ indicates the distribution of Foraminifera (Ref. 2) and 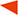 indicates the distribution of brackish fish (Ref. 3).

**Figure 3:**
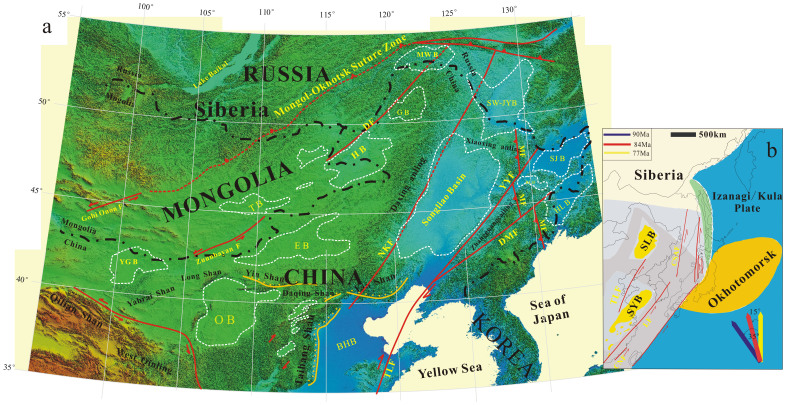
Location and tectonic map of the Songliao Basin. (a): Basic topographic map of Songliao Basin, separated from Siberia by the Mongol–Okhotsk suture zone, and bounded by the Yinshan–Yanshan belt on the south and by the Daxing'anling belt on the east (B = basin; F = fault). The closed dotted white lines indicate Mesozoic–Cenozoic basins. YGB = Yinggen Basin; OB = Ordos Basin; EB = Erlian Basin; TB = Tamsag Basin; BHB = Bohai Basin; HB = Hailar basin; GB = Genhe Basin; MWB = Mohe Baisn; SLB = Songliao Basin; SW-JYB = Sunwu-Jiaying Basin; HLB = Hulin Basin; SJB = Sanjiang Basin; BLB = Boli Basin; DMF = Dunhua–Mishan Fault; MF = Mudanjiang Fault; DF = Derbugan Fault; YYF = Yilan–Yitong Fault; NKF = Nenjiang–Kailu Fault; TLF = Tanlu Fault. (Modified after Wang et al., 2013b (Ref. 29). (b), The drifting history of the Izanagi/Kula Plate since 90 Ma (modified after Yang, 2013 and Norton, 2007). SLB = Songliao Basin; SYB = Subei-Yellow Sea Basin. CNF = Changle-Nanao Fault; CSAF = Central Sikhote-Alin Fault; LF = Lishui Fault; TLF = Tanlu Fault. The Okhotomorsk Block moved northward along the East Asian margin, due to the change from N35°W to N15°W in Izanagi motion direction. The northeastward oblique motion of the Okhotomorsk Block along the transform zone at the Asian margin resulted in a sinistral strike-slip fault system of several tens of kilometers wide in North China (Ref. 22).
