# Supplementary material for: Seawater Incursion Events in a Cretaceous Paleo-lake Revealed by Specific Marine Biological Markers
Source: Sci Rep. 2015 May 7;5:9508. doi: 10.1038/srep09508 (PMC4423240; doi:10.1038/srep09508)
Supplement: Supplementary Information — Supplementary [file srep09508-s1.doc]

**Seawater Incursion Events in a Cretaceous Paleo-lake Revealed by Specific Marine Biological Markers**

**J.F. Hu^1^, P.A. Peng^1^*, M.Y. Liu^2^, D.P. Xi^2^, J. Z. Song^1^, X.Q. Wan^2^ and C.S. Wang^2^**

^1^State Key Laboratory of Organic Geochemistry, Guangzhou Institute of Geochemistry, Chinese Academy of Sciences, Guangzhou, 510640, P R China

^2^State Key Laboratory of Biogeology and Environmental Geology, China University of Geosciences, Beijing 100083, China

*e-mail: pinganp@gig.ac.cn

Supplementary :

**TOC and carbon-isotope analysis**

Solvent-rinsed core rock fragments and cuttings were first ground to 80 meshes, after which 5% hydrochloride was added to a portion of each sample (to eliminate the inorganic carbonates) and subsequently rinsed with deionized water (to remove salts) and dehydrated in oven at 60 ^o^C. The carbonate-free samples were then analyzed (on a Vario EL-III elemental analyzer) to determine weight percent of organic carbon. Duplicate analyses of every sample were run and the mean value of the two measurements is reported here. Replicate analysis of one sample (*n* =5) gave a 1 ơ  precision of ± 0.02 %.

The δ^13^C_org_ analyses were performed on the carbonate-free rock sample, using a DELTA^plus^ XL mass spectrometer. Results (reported as parts per mil (‰)) were estimated as follows:

δ(‰) = $\frac{\text{R}_{\text{sample}}-\text{R}_{\text{reference}}}{\text{R}_{\text{reference}}}\times1000$,

where δ (‰) = δ^13^C(‰), and R_sample_ and R_reference_ are the isotopic ratios of the sample and the reference, respectively. In the case of carbon, the reference is Peedee belemnite (PDB). Reproducibility, based on triplicate analyses of a sample, was ± 0.2‰ for δ^13^C.
